# Assortativity and leadership emerge from anti-preferential attachment in heterogeneous networks

**DOI:** 10.1038/srep21297

**Published:** 2016-02-18

**Authors:** I. Sendiña-Nadal, M. M. Danziger, Z. Wang, S. Havlin, S. Boccaletti

**Affiliations:** 1 Complex Systems Group & GISC, Universidad Rey Juan Carlos, 28933 Móstoles, Madrid, Spain; 2 Center for Biomedical Technology, Universidad Politécnica de Madrid, 28223 Pozuelo de Alarcón, Madrid, Spain; 3 Department of Physics, Bar Ilan University, Ramat Gan 52900, Israel; 4 School of Automation, Northwestern Polytechnical University, Xi’an 710072, China; 5 Interdisciplinary Graduate School of Engineering Sciences, Kyushu University, Fukuoka, 816-8580, Japan; 6 CNR- Institute of Complex Systems, Via Madonna del Piano, 10, 50019 Sesto Fiorentino, Florence, Italy; 7 The Italian Embassy in Israel, 25 Hamered st., 68125 Tel Aviv, Israel

## Abstract

Real-world networks have distinct topologies, with marked deviations from purely random networks. Many of them exhibit degree-assortativity, with nodes of similar degree more likely to link to one another. Though microscopic mechanisms have been suggested for the emergence of other topological features, assortativity has proven elusive. Assortativity can be artificially implanted in a network via degree-preserving link permutations, however this destroys the graph’s hierarchical clustering and does not correspond to any microscopic mechanism. Here, we propose the first generative model which creates heterogeneous networks with scale-free-like properties in degree and clustering distributions and tunable realistic assortativity. Two distinct populations of nodes are incrementally added to an initial network by selecting a subgraph to connect to at random. One population (the followers) follows preferential attachment, while the other population (the potential leaders) connects via *anti-preferential* attachment: they link to lower degree nodes when added to the network. By selecting the lower degree nodes, the potential leader nodes maintain high visibility during the growth process, eventually growing into hubs. The evolution of links in Facebook empirically validates the connection between the initial anti-preferential attachment and long term high degree. In this way, our work sheds new light on the structure and evolution of social networks.

Networks with scale-free(SF)-like degree distributions represent a wide range of systems^1^,^2^,^3^,^4^,^5^,^6^. The topology of real-world networks (RWNs) often features deviations from a pure power-law distribution^2^ 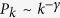, together with hierarchical clustering^7^ 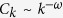. One ubiquitous feature of many RWNs is degree-degree correlations: two nodes are more likely to be linked to one another if they are of similar (assortative) or dissimilar (disassortative) degree. Assortativity is generally found in social and collaboration RWNs, while disassortativity is common in technological and biological RWNs^8^,^9^.

SF networks have been studied in the context of generative models, and simple rules relating to the formation of new links have been shown to lead to power-law degree distributions with non-hierarchical^10^,^11^ and hierarchical^12^,^13^,^14^,^15^,^16^,^17^,^18^ traits. Static SF network models^19^ have also been proposed with controlled assortativity^20^,^21^, and growing SF networks have been studied with assortative^22^,^23^,^24^,^25^,^26^, disassortative^10^,^27^ and both types^11^ of degree mixing.

In particular, a wide range of RWNs features assortativity^28^, including online social^29^, and neural^30^,^31^ networks. As it reflects a basic *birds of a feather flock together* property, it is not surprising that it is so ubiquitous. Rather, what is really surprising is that the contributions of different nodes to the graph assortativity level *r* strongly depend on the degree. Decomposing the assortativity spectrum, one can indeed describe the *local* assortativity or assortativeness^32^ 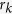 of each set of nodes with a given degree *k* (see the Methods section). Many RWNs have a pronounced local maximum in 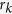 located near (but above) the average degree 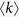. In social networks such a feature even appears to be generic, while in technological and biological networks the maximum is less pronounced or even entirely absent. In Fig. 1 we show the qualitative difference in the inherent patterns of 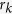 between typical social networks (the friendship structure of Facebook users^29^, Fig. 1a, and the Authors’ collaboration graph from the arXiv’s Astrophysics section^33^,^34^,^35^, Fig. 1d) and a technological one (the flights connecting the 500 busiest commercial airports in the United States^36^, Fig. 1b).

## Results

### Empirical observations

**Figure 1:**
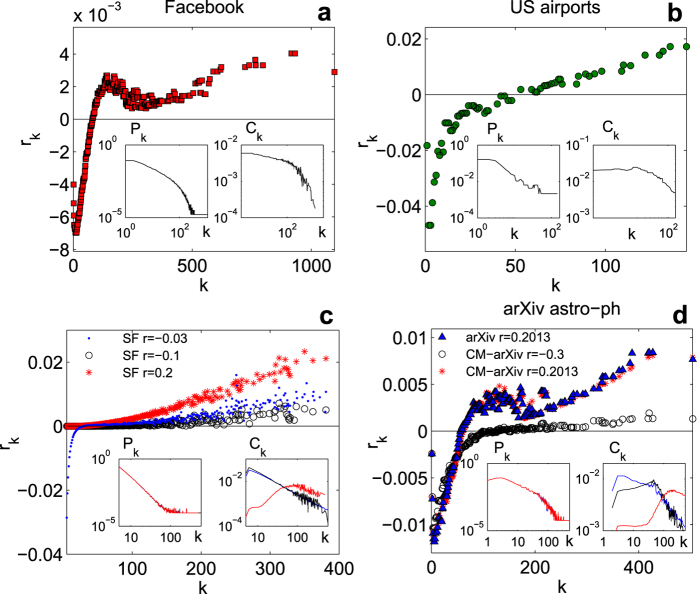
Local assortativity *r*_*k*_ *vs.* the node degree *k* for real^28^ and artificial networks. (**a**) Data from friendships of Facebook users^29^ 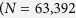, 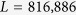, 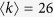, 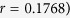. (**b**) Network of the 500 busiest commercial airports in the United States^36^. A tie exists between two airports if a flight was scheduled in 2002 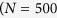, 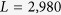, 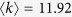, 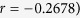. (**c**) Random SF networks 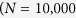, 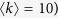 with almost neutral 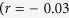, blue dots), disassortative 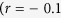, black circles) and assortative 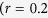, red stars) mixing. (**d**) The Authors’ collaboration graph from the arXiv’s Astrophysics section^33^ 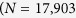, 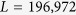, 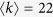, 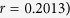. Together with the real data (blue triangles), 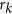 is reported for a configuration model (CM) reproducing the real degree sequence, after classical permutation methods have been applied, imposing the same *r* value observed in the real network (red stars) and a negative 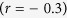 value (black circles). Insets in panels (**a**–**d**) show the log-log plots of the degree distributions 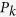 and clustering coefficient 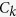.

The way traditional methods imprint assortativity into pre-generated networks is via degree-preserving link permutations^9^,^37^. This approach yet presents a number of problems. On the one hand, generating a graph with an ad-hoc imprinted SF distribution (Fig. 1c) and then rewiring connections *does not* yield the observed pattern of local assortativity, on the other hand, even starting from a configuration model (CM) retaining the original degree distribution^19^, this procedure is only able to reproduce the real assortativity pattern at the expense of destroying the other significant features, such as the hierarchical inherent structure of clustering (Fig. 1d and its bottom-right inset). This indicates that the systemic mechanisms leading to the emergence of degree-correlation have a special signature, which is not captured when generating assortativity artificially, i.e., *ex post facto*.

**Figure 2:**
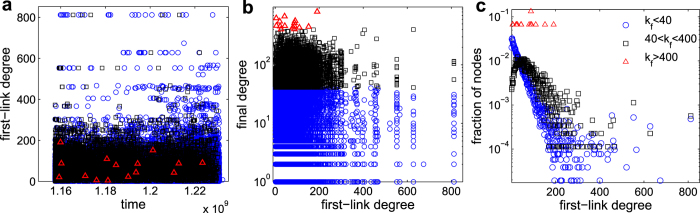
Nodes’ selection mechanisms of their initial neighbors in RWNs. The Facebook network analyzed in Fig. 1a.(**a**) Degree of the nodes chosen as first connections by those nodes whose final (i.e. at the end of the growth process) degree 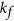 is low 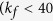, blue circles), high 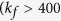, red triangles), and intermediate 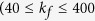, black squares). The reported values are from the largest connected component of the Facebook network of Fig. 1a formed only by those edges that are time-stamped 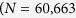, 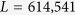, 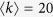, 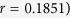. (**b**) Log-linear plot of of the final degree 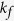 of each node (labeled according to the legend in Fig. 2c) as a function of the degree of its first connection. (**c**) Log-linear plot of the fraction of high (red triangles), medium (black squares) and low (blue circles) degree nodes establishing their first link with a node of a given degree.

Further striking evidence comes to light from a deeper analysis of social RWNs: in some cases the final leaders (i.e. the nodes that, at the end of the process, do acquire a leading role in terms of their degree) actually behave *anti-preferentially* when entering the network. In Fig. 2, the Facebook network of Fig. 1a is examined, and one sees that, plotting the degree of the first linked node as a function of time, those nodes eventually becoming the network’s leaders (i.e. the final hubs, red triangles) tend initially (at the moment at which they start forming part of the network) to link existing nodes with low degree values (Fig. 2a). This is clearer from Fig. 2b where the final degree 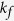 achieved by a given node, labeled as a red triangle 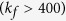, a black square 
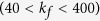
 or a blue circle 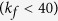, is compared to the degree of its first neighbor at the time that node entered the network. A straightforward statistical analysis of the data shows in Fig. 2c that indeed the fraction of final hubs forming initial connections with nodes of low-medium degrees is far larger than that of the nodes which ultimately acquire intermediate and low degrees.

### The generative model

Following the empirical observation in Fig. 2 of a nexus between initial anti-preferential attachments and long-term high degrees, we propose a generative model which creates SF-like networks with tunable global assortativity and realistic local assortativity patterns, while also reproducing the hierarchical structure of the network’s clustering. The model reflects a microscopic mechanism for a *struggle for leadership* between two competing populations of nodes: type I nodes (acting as *followers* and selecting connections so that a preferential attachment rule spontaneously emerges^10^) and type II nodes (acting as *potential leaders*, i.e. adopting anti-preferential behavior which leads them to prefer lower degree nodes for the establishment of their initial links).

**Figure 3:**
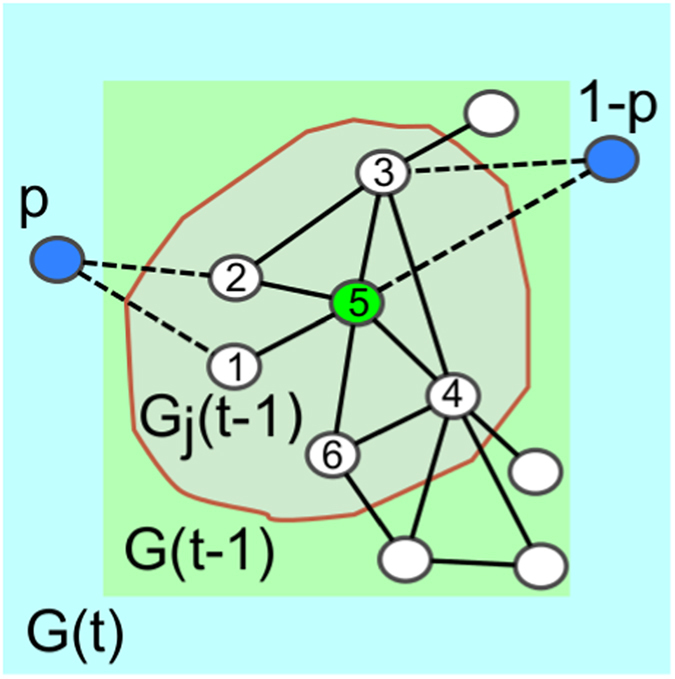
The network growth process. At time *t*, the graph 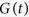 is updated with a new node (blue circle) which forms *m* connections (in the example 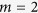, dashed lines) within the subgraph 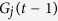 with a probability *p* to the lowest degree nodes (nodes 1 and 2) or with probability 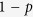 at random (nodes 3 and 5). The subgraph 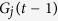 is composed of a randomly chosen node *j* (node 5, green circle) and its nearest neighbors at time 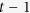.

Under such a mechanism, a network of *N* nodes is created by sequentially adding units to an initial clique of 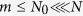 vertices. The growing process occurs at discrete times: at each time step 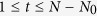 a new node enters the graph, and forms *m* links with existing nodes according to an attachment rule that is illustrated schematically in Fig. 3 and summarized as follows:

1. An anchor node *j* is selected uniformly at random from the nodes existing at time 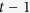.
2. The subgraph 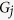 composed of node *j* and all other nodes that are at distance less than or equal to 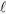 from *j* is examined.
3. With probability 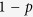, the new node behaves as a *follower* (type I): it selects *m* nodes from 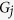 uniformly at random, and links to them. With probability *p*, the new node behaves instead as a *potential leader* (type II): it forms links with the *m* lowest degree nodes in 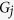.

The parameter 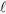 is defined as the so called *penetration depth*, i.e. the extent of local information (around the anchor *j*) accessible to the entering node. In the following, we set 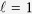, so that 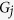 is the subgraph containing *j* and all its nearest neighbors. Once 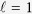 is set, the model is uniquely determined by two parameters: the average degree 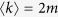 and *p*, the fraction of type II nodes. In the absence of *potential leaders* 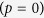, the growth of the resulting network exhibits emergent preferential attachment and hierarchical clustering^10^: the 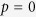 case produces a pure SF network with degree distribution^2^,^4^ 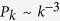, and with additional hierarchical SF clustering^7^ 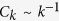. This is actually due to the so called *friendship paradox*^38^, stating that, averaged across the network, the neighbors of a node *i* will always have a higher average degree than 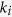. Since, indeed, the number of subgraphs 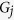 in which a node *i* appears is equal to 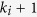, higher degree nodes will tend to naturally receive more and more links. It is important to note that this preferential behavior is in fact, emergent: the entering nodes do not require global knowledge of the degree levels in the system, nor any explicit preference for high degree nodes. In that sense, preferential attachment can be viewed as a kind of null behavior in which the rate of growth increases with size, as the analogous Yule process is understood in evolutionary dynamics^39^,^40^.

**Figure 4:**
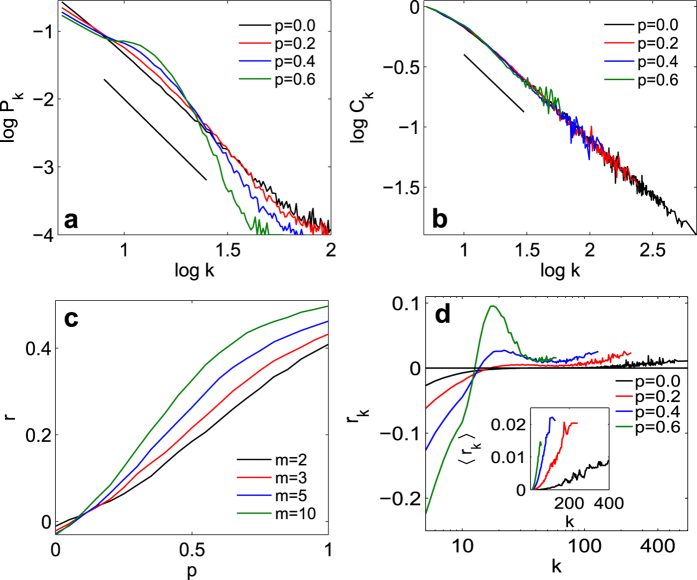
Emergent topology in the generated network. (**a**) Normalized degree distribution *P*_*k*_ (log_10_ scale) *vs.* the logarithm (base 10) of *k*, and (**b**) 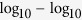 plot of 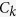 *vs. k*, for 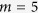 and different values of the probability *p* (see legend for color-coding). (**c**) Assortativity coefficient *r vs. p*, for different values of *m* (see legend for color-coding). (**d**) Log-linear plot of the local assortativity 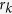 (main panel) and average local assortativity 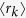 (inset) *vs. k*, for 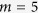 and several values of *p* (see legend for color-coding). In all cases, 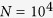, 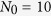, and each point refers to an ensemble average over 20 network realizations. As a guide for the eyes, the straight lines in (**a**,**b**) stay for the functions 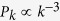 and 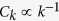, respectively.

When instead the population is split (with some nodes following the null preferential attachment, and some others linking in an anti-preferential manner), the local assortativity pattern shown in Fig. 1a, characterizing social systems, emerges. Namely, the contribution to assortativity from nodes of degree *k i*) increases with *k* from 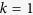 to a local maximum located just above the average degree, *ii*) decreases to a subsequent local minimum, and then *iii)* increases again as 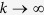, i.e. qualitatively reproducing the generic tendency observed in social RWNs, which is only captured in random generated networks with artificially induced assortativity at the expense of obliterating the graph’s clustering traits. The results of the model are summarized in Fig. 4. As *p* increases, the degree distribution of the resulting network deviates more and more from a pure SF configuration (Fig. 4a), but at the same time the hierarchical clustering traits are entirely preserved (Fig. 4b). The generated network is actually endowed with a fully controllable and tunable level of global assortativity *r* (as a function of *m*, as shown in Fig. 4c), while, more remarkably, the assortativity local pattern is fully reproduced (Fig. 4d).

### Analytical description

We next move toward giving a more analytic description of the motivations and roots underlying the proposed model and the observed, emergent phenomena. We start by noting that links in this model are undirected, and this leads to a symmetry of interpretations: one can describe the type II nodes as preferring low-degree units (as it is described in our generative model), or one can state that low-degree nodes are more likely to create links with type II newcomers. The second interpretation is actually in line with what arises from recent sociological studies, which indeed indicate that people are limited in the number of relationships they can maintain over time (with the exact number of maximal relationships being an open question). Starting from the seminal works by Dunbar^41^,^42^, the limitations on the number of active social connections have been extensively studied and empirical support from online social networks has also been adduced^43^. In the present case, the emergence of positive assortativity is associated with the interplay of two mechanisms: an innate preferential attachment (resulting from nodes that nonhierarchically form connections with a pre-existing growing structure) and a limited ability of human beings to maintain many relationships.

By comparing the average contribution of assortativity per node of degree *k*, 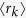, and the total contribution of nodes of degree *k*, 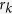, one can actually understand the origin of the peak in the local assortativity. The average contribution for nodes of degree *k* increases monotonically with *k* (inset of Fig. 4d). However, the frequency of nodes decreases monotonically with *k* in pure scale-free networks (Fig. 4a). With the introduction of type II nodes, lower-medium degree nodes become more frequent, as observed in Fig. 4a for 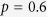, even though an overall scale-free-like degree distribution is maintained. The combination of more-common than expected medium degree nodes and per-node contribution to assortativity that increases with *k* leads to the characteristic bump observed in the model and the data.

As the network’s growth proceeds, type II nodes actually tend to develop a higher degree on average. This is because new links are obtained with probability


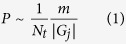


where 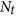 is the number of nodes in the system at time *t* and 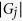 is the size of the neighborhood of the subgraph of a given anchor node *j*. By choosing anchor nodes with small 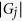 (low degree), type II nodes actually increase their likelihood of being linked from future, incoming, nodes. Because this increased likelihood can be understood as type II nodes “placing themselves” in smaller neighborhoods so that they are more likely to be linked to than when chosen at random, we understand this advantage as a kind of improved visibility to the linking process.

**Figure 5:**
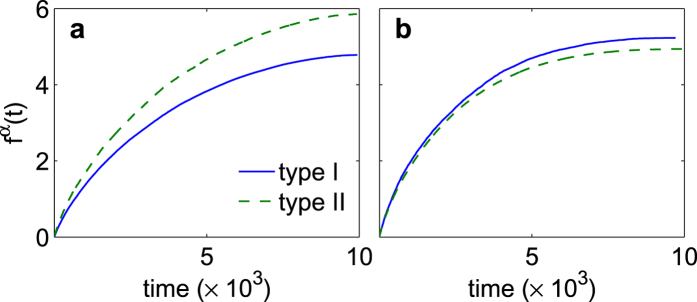
Emergence of leadership during the growth process. Average increased degree (the degree acquired after nodes have first appeared in the graph, vertical axes) as a function of time (horizontal axis), for type I (followers) and type II (potential leaders) nodes, and for (**a**) 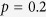, and (**b**) 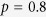. See the Methods section for the explanation on how the reported values are calculated. Panels report the average increased degree 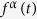 of the nodes of different types (*α* = type I or II), after having been in the system for *t* steps. 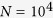, 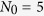 and 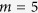. Color and line style codes are defined in the legend of panel (**a**).

In fact, one can measure the number of neighbors at time *t* for each node type as described in the Methods section. The results are shown in Fig. 5, and point to the emergence of leadership of type II nodes at low values of *p* (Fig. 5a). At intermediate values of *p* (not shown) no significant differences are observed between the two nodes’ populations in the way the average increased degree evolves in time. Only at large *p* values (Fig. 5b), where anti-preferential nodes are vastly predominant in number the trend is actually reversed and type I nodes (the followers) now seem to be favored in attracting connections. Such a latter situation corresponds however to a rather homogeneous network, where a SF-like distribution is no longer observed (see Fig. 4 for comparing the large deviations in the degree distribution already observed at 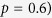.

## Discussion

In summary, assortativity, hierarchical structure and fat-tailed degree distributions (well-approximated by power laws) are structural features manifested almost ubiquitously by RWNs, and until now no model had ever linked their emergence with microscopic growing assumptions. Furthermore, these features have a fundamental role in determining many relevant processes, and/or regulating the network’s dynamics and functioning. Guided by the empirical observation of the growth of the friendship network of Facebook users, we have shown how the combination of preferential and anti-preferential attachment mechanisms acting together in the same generative model (via two distinct node populations), leads to the growth of heterogeneous networks with modified scale-free properties and tunable realistic assortativity, while maintaining the hierarchical clustering. Both our analytical predictions and numerical results indicate that networks constructed in this way match the patterns of local assortativity measured in real-world graphs. By presenting the first generative model with tunable assortativity, this work sheds new light on the structure and evolution of social networks, and counterintuitively suggests that anti-preferential attachment is a mechanism adopted by a fraction of the nodes during the network’s growth, as a strategy for increasing their own leadership.

## Methods

### Local assortativity/assortativeness

In a network with *N* nodes, *L* links and degree distribution 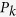, the local assortativity or assortativeness^32^ 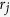 is defined as the contribution of each node to the network assortativity *r* and it is calculated as


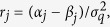


with 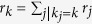 being the total of the local assortativity values of nodes with a given degree *k* such that 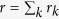. In the above expression, 
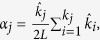
 and 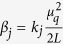, being 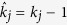 the remaining degree of node *j*, 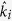 the remaining degrees 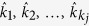 of the 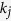 nodes connected to node *j*, and 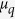 and 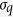 the first and second moments of the remaining degree distribution 
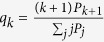
.

### Measuring the average degree of each node type

In order to compare the average degree of the two node populations as the model evolves, we label each node uniquely by the step in which it entered the network. This way, at time *t*, every node *i* will have *m* neighbors with indices 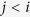, and 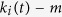 neighbors with indices 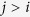. To compare the degree growth rates of type I and type II nodes, we need to measure the characteristic time for new links to form. To do so, we consider the set of differences in index values, 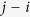, for each neighbor which linked to *i* at step *j*


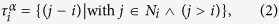


with 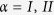 designating the node type and 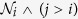 the neighborhood of *i*. Combining these sets for all nodes of each type, one obtains the non-unique set


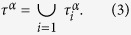


Using Eq. (3), one can measure the expected number of neighbors (after 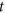 steps) for each node type via


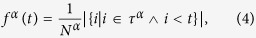


where 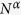 is the total number of nodes of type *α*. Thus 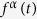 provides the average number of new neighbors 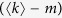 that a node of type *α* will acquire after *t* steps.

## Additional Information

**How to cite this article**: Sendiña-Nadal, I. *et al.* Assortativity and leadership emerge from anti-preferential attachment in heterogeneous networks. *Sci. Rep.* **6**, 21297; doi: 10.1038/srep21297 (2016).
